# (*E*)-2-[1-(1-Benzothio­phen-3-yl)ethyl­idene]hydrazinecarbothio­amide methanol hemisolvate

**DOI:** 10.1107/S160053680801297X

**Published:** 2008-05-10

**Authors:** Safa’a Fares Kayed, Yang Farina, Mohammad Kassim, Jim Simpson

**Affiliations:** a School of Chemical Sciences and Food Technology, Faculty of Science and Technology, Universiti Kebangsaan Malaysia, 43600 UKM Bangi, Selangor, Malaysia; b Department of Chemistry, University of Otago, P.O. Box 56, Dunedin, New Zealand

## Abstract

The asymmetric unit of the title compound, C_11_H_11_N_3_S_2_·0.5CH_4_O, contains four thio­semicarbazone mol­ecules and two methanol solvent mol­ecules. Each hydrazinecarbothio­amide mol­ecule adopts an *E* configuration with respect to the C=N double bond and is stabilized by an intra­molecular N—H⋯N hydrogen bond, resulting in an *S*(5) ring motif. In the crystal structure, an extensive network of N—H⋯O, N—H⋯N, O—H⋯S and N—H⋯S hydrogen bonds and weak C—H⋯O, C—H⋯N and C—H⋯S contacts together with an S⋯S [3.5958 (14) Å] and a C—H⋯π inter­action form a three-dimensional network.

## Related literature

For related structures, see: de Lima *et al.* (2002 ▶); Işık *et al.* (2006 ▶). For reference structural data, see: Allen *et al.* (1987 ▶). For graph-set analysis of hydrogen bonding, see: Bernstein *et al.*, (1995 ▶).
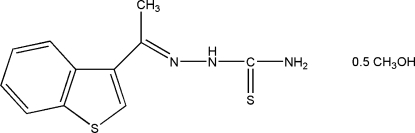


## Experimental

### 

#### Crystal data

C_11_H_11_N_3_S_2_·0.5CH_4_O
*M* *_r_* = 265.38
Monoclinic, 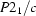

*a* = 18.9438 (12) Å
*b* = 17.7076 (11) Å
*c* = 15.4145 (10) Å
β = 107.238 (3)°
*V* = 4938.5 (5) Å^3^
*Z* = 16
Mo *K*α radiation
μ = 0.41 mm^−1^
*T* = 92 (2) K
0.31 × 0.20 × 0.13 mm

#### Data collection

Bruker APEXII CCD area-detector diffractometer
Absorption correction: multi-scan (*SADABS*; Bruker, 2006 ▶) *T*_min_ = 0.777, *T*_max_ = 0.947
65034 measured reflections
10871 independent reflections
8171 reflections with *I* > 2σ(*I*)
*R*_int_ = 0.086

#### Refinement

*R*[*F* ^2^ > 2σ(*F* ^2^)] = 0.067
*wR*(*F* ^2^) = 0.203
*S* = 1.03
10871 reflections
655 parameters
12 restraints
H atoms treated by a mixture of independent and constrained refinement
Δρ_max_ = 1.71 e Å^−3^
Δρ_min_ = −2.31 e Å^−3^

### 

Data collection: *APEX2* (Bruker, 2006 ▶); cell refinement: *APEX2* and *SAINT* (Bruker, 2006 ▶); data reduction: *SAINT* (Bruker, 2006 ▶); program(s) used to solve structure: *SHELXS97* (Sheldrick, 2008 ▶) and *TITAN2000* (Hunter & Simpson, 1999 ▶); program(s) used to refine structure: *SHELXL97* (Sheldrick, 2008 ▶) and *TITAN2000*; molecular graphics: *SHELXTL* (Sheldrick, 2008 ▶), *ORTEP-3* (Farrugia, 1997 ▶) and *Mercury* (Macrae *et al.*, 2006 ▶); software used to prepare material for publication: *SHELXL97*, *enCIFer* (Allen *et al.*, 2004 ▶) and *PLATON* (Spek, 2003 ▶).

## Supplementary Material

Crystal structure: contains datablocks global, I. DOI: 10.1107/S160053680801297X/hb2725sup1.cif

Structure factors: contains datablocks I. DOI: 10.1107/S160053680801297X/hb2725Isup2.hkl

Additional supplementary materials:  crystallographic information; 3D view; checkCIF report

## Figures and Tables

**Table 1:** Hydrogen-bond geometry (Å, °)

*D*—H⋯*A*	*D*—H	H⋯*A*	*D*⋯*A*	*D*—H⋯*A*
N3*A*—H3*N*2⋯N1*A*	0.843 (10)	2.29 (4)	2.641 (4)	105 (3)
N3*B*—H3*N*3⋯N1*B*	0.838 (10)	2.21 (4)	2.617 (4)	110 (3)
N3*C*—H3*N*5⋯N1*C*	0.842 (10)	2.22 (5)	2.543 (4)	102 (4)
N3*D*—H3*N*7⋯N1*D*	0.841 (10)	2.29 (5)	2.643 (4)	105 (4)
N3*A*—H3*N*1⋯S2*B*^i^	0.839 (10)	2.500 (14)	3.325 (3)	168 (4)
N3*B*—H3*N*4⋯S2*A*^ii^	0.839 (10)	2.62 (2)	3.367 (3)	149 (4)
N2*B*—H2*NB*⋯S2*A*^iii^	0.839 (10)	2.585 (13)	3.412 (3)	169 (4)
N3*C*—H3*N*6⋯S2*A*^iv^	0.840 (10)	2.76 (4)	3.352 (3)	129 (4)
N3*C*—H3*N*6⋯N3*B*^v^	0.840 (10)	2.72 (3)	3.468 (5)	149 (5)
N2*C*—H2*NC*⋯S2*C*^vi^	0.842 (10)	2.60 (2)	3.392 (3)	158 (4)
N3*D*—H3*N*8⋯S2*A*^iv^	0.840 (10)	2.738 (15)	3.563 (3)	168 (4)
N3*D*—H3*N*7⋯O1*S*	0.841 (10)	2.26 (3)	2.967 (6)	142 (4)
O1*S*—H1*S*⋯S2*C*^vi^	0.84	2.64	3.471 (5)	169
O2*S*—H2*S*⋯S2*D*^vii^	0.84	2.97	3.389 (6)	113
C3*A*—H3*A*⋯S2*A*^iv^	0.95	2.97	3.762 (4)	142
C10*A*—H10*A*⋯S2*B*^viii^	0.98	2.91	3.613 (3)	129
C2*B*—H2*B*⋯O1*S*^ix^	0.95	2.36	3.282 (7)	163
C2*S*—H2*S*1⋯N2*D*^vii^	0.98	2.74	3.266 (9)	115
C10*B*—H10*F*⋯C_g_^x^	0.98	2.91	3.594 (4)	129

Symmetry codes: (i) 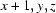; (ii) 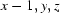; (iii) 
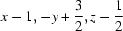
; (iv) 
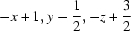
; (v) 
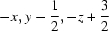
; (vi) 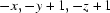; (vii) 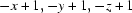; (viii) 
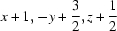
; (ix) 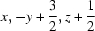; (x) 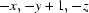. *Cg* is the centroid of C1*B*–C6*B.*

**Fig. 1.**
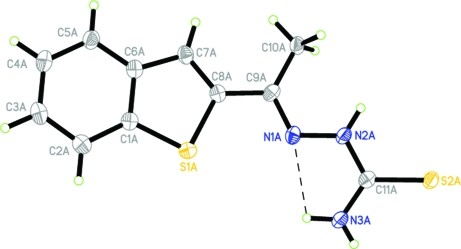
The structure of molecule A in (I) showing 50% probability displacement ellipsoids for the non-H atoms. The intramolecular N—H···N hydrogen bond is drawn as a dashed line.

**Fig. 2.**
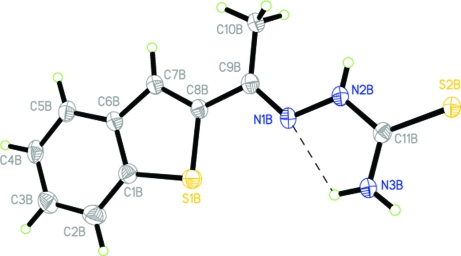
The structure of molecule B in (I) showing 50% probability displacement ellipsoids for the non-H atoms. The intramolecular N—H···N hydrogen bond is drawn as a dashed line.

**Fig. 3.**
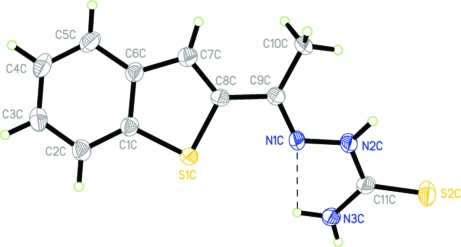
The structure of molecule C in (I) showing 50% probability displacement ellipsoids for the non-H atoms. The intramolecular N—H···N hydrogen bond is drawn as a dashed line.

**Fig. 4.**
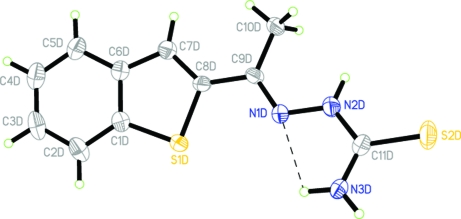
The structure of molecule D in (I) showing 50% probability displacement ellipsoids for the non-H atoms. The intramolecular N—H···N hydrogen bond is drawn as a dashed line.

**Fig. 5.**
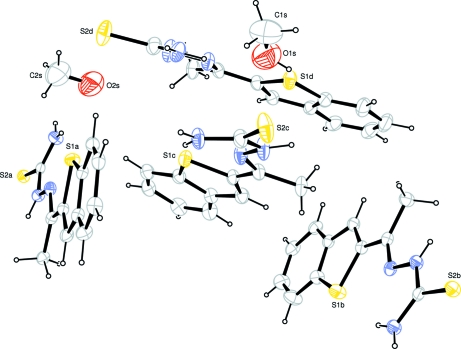
The asymmetric unit of (I) with intermolecular hydrogen bonds drawn as dashed lines.

**Fig. 6.**
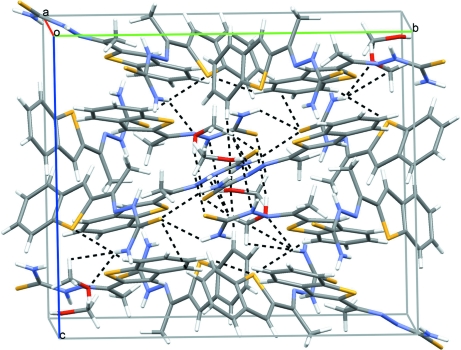
Crystal packing of (I) viewed down the a axis with hydrogen bonds drawn as dashed lines.

## supplementary crystallographic information

### Comment

Structures similar to the title compound, (I), have been reported including the thiosemicarbazone derived from 2-acetylthiophene (Lima *et al.*, 2002); and a pryazoline derivative (Işık *et al.*, 2006).

The asymmetric unit of (I) contains four molecules, labeled A—D (Figs 1—4) and two methanol solvate molecules, with the complete assemblage shown in Fig. 5.  Each molecule adopts an *E* configuration with respect to the C=N bond and bond distances and angles are normal (Allen *et al.*, 1987). Intramolecular N3—H···N1 hydrogen bonds (Table 1) form between each of the the NH_2_ groups and the imine N atoms generating S(5) ring motifs (Bernstein *et al.*, 1995). These contribute to the planarity of the molecules.

In the crystal of (I), N2—H2···S2 hydrogen bonds generate centrosymmetric *R*^2^_2_(8) rings in molecules A—C. For molecule D, C2S—H2S1···S2D hydrogen bonding to a methanol solvate within the asymmetric unit obviates such an interaction. Other N—H···O, N—H···N, O—H···S and N—H···S hydrogen bonds together with weak C—H···O, C—H···N and C—H···S contacts, an S···S interaction (*d*(S1D···S1B^i^) = 3.5958 (14) Å; i = *x*, 3/2 - *y*, -1/2 + *z*) and a C10B—H10F···*Cg*^ii^ interaction (ii = -*x*, 1 - *y*, -*z*; *Cg* is the centroid of the C1B···C6B ring) form a complex three dimensional network (Fig 6).

### Experimental

A 1:1 mixture of 2-acetylbenzothiophene and thiosemicarbazide was heated under reflux in ethanol for 2 h. The solid product which separated upon cooling was filtered and recrystallized from a 1:1 mixture of acetonitrile and methanol to afford colourless, blocks of (I) in 68% yield (m.p. 483–485 K).

### Refinement

The C-bound H atoms were placed geometrically (C—H = 0.95-0.98Å) and refined as riding with *U*_iso_=1.2*U*_eq_(C) of 1.5U_eq_(methyl C).

The N-bound H atoms were located in a difference map and refined with a distance restraint of N—H = 0.84 (1) Å, and with *U*_iso_(H) = 1.2*U*_eq_(N) (carrier).

The highest residual electron density peak is 0.07 Å from O2S and the deepest hole is 0.04Å from C2S suggesting the possibility of unresolved disorder in this methanol solvate molecule.

### Crystal data

**Table:**

C_11_H_11_N_3_S_2_·0.5(CH_4_O)	*F*_000_ = 2224
*M**_r_* = 265.38	*D*_x_ = 1.428 Mg m^−^^3^
Monoclinic, *P*2_1_/*c*	Mo *K*α radiation λ = 0.71073 Å
Hall symbol: -P 2ybc	Cell parameters from 8980 reflections
*a* = 18.9438 (12) Å	θ = 2.3–25.6º
*b* = 17.7076 (11) Å	µ = 0.42 mm^−^^1^
*c* = 15.4145 (10) Å	*T* = 92 (2) K
β = 107.238 (3)º	Block, colourless
*V* = 4938.5 (5) Å^3^	0.31 × 0.20 × 0.13 mm
*Z* = 16	

### Data collection

**Table:**

Bruker APEXII CCD area-detector diffractometer	10871 independent reflections
Radiation source: fine-focus sealed tube	8171 reflections with *I* > 2σ(*I*)
Monochromator: graphite	*R*_int_ = 0.086
*T* = 92(2) K	θ_max_ = 27.1º
ω scans	θ_min_ = 1.1º
Absorption correction: multi-scan(SADABS; Bruker, 2006)	*h* = −24→24
*T*_min_ = 0.777, *T*_max_ = 0.947	*k* = −22→22
65034 measured reflections	*l* = −19→18

### Refinement

**Table:**

Refinement on *F*^2^	Secondary atom site location: difference Fourier map
Least-squares matrix: full	Hydrogen site location: inferred from neighbouring sites
*R*[*F*^2^ > 2σ(*F*^2^)] = 0.067	H atoms treated by a mixture of independent and constrained refinement
*wR*(*F*^2^) = 0.203	*w* = 1/[σ^2^(*F*_o_^2^) + (0.1068*P*)^2^ + 8.4506*P*] where *P* = (*F*_o_^2^ + 2*F*_c_^2^)/3
*S* = 1.03	(Δ/σ)_max_ = 0.001
10871 reflections	Δρ_max_ = 1.71 e Å^−^^3^
655 parameters	Δρ_min_ = −2.30 e Å^−^^3^
12 restraints	Extinction correction: none
Primary atom site location: structure-invariant direct methods	

### Special details

**Table:**

Geometry. All e.s.d.'s (except the e.s.d. in the dihedral angle between two l.s. planes) are estimated using the full covariance matrix. The cell e.s.d.'s are taken into account individually in the estimation of e.s.d.'s in distances, angles and torsion angles; correlations between e.s.d.'s in cell parameters are only used when they are defined by crystal symmetry. An approximate (isotropic) treatment of cell e.s.d.'s is used for estimating e.s.d.'s involving l.s. planes.
Refinement. Refinement of *F*^2^ against ALL reflections. The weighted *R*-factor *wR* and goodness of fit *S* are based on *F*^2^, conventional *R*-factors *R* are based on *F*, with *F* set to zero for negative *F*^2^. The threshold expression of *F*^2^ > σ(*F*^2^) is used only for calculating *R*-factors(gt) *etc*. and is not relevant to the choice of reflections for refinement. *R*-factors based on *F*^2^ are statistically about twice as large as those based on *F*, and *R*- factors based on ALL data will be even larger.

### Fractional atomic coordinates and isotropic or equivalent isotropic displacement parameters (Å^2^)

**Table:**

	*x*	*y*	*z*	*U*_iso_*/*U*_eq_	
C1A	0.40620 (18)	0.50684 (18)	0.8528 (2)	0.0201 (7)	
C2A	0.36631 (19)	0.44650 (19)	0.8029 (2)	0.0241 (7)	
H2A	0.3746	0.4300	0.7480	0.029*	
C3A	0.3144 (2)	0.41176 (19)	0.8364 (2)	0.0254 (7)	
H3A	0.2872	0.3700	0.8046	0.030*	
C4A	0.3010 (2)	0.4368 (2)	0.9163 (3)	0.0263 (8)	
H4A	0.2643	0.4125	0.9370	0.032*	
C5A	0.3402 (2)	0.4960 (2)	0.9651 (3)	0.0252 (7)	
H5A	0.3309	0.5124	1.0193	0.030*	
C6A	0.39438 (18)	0.53217 (18)	0.9341 (2)	0.0212 (7)	
S1A	0.47650 (5)	0.55720 (5)	0.82797 (6)	0.0235 (2)	
C7A	0.44278 (18)	0.59319 (19)	0.9744 (2)	0.0224 (7)	
H7A	0.4422	0.6176	1.0291	0.027*	
C8A	0.48976 (18)	0.61239 (18)	0.9258 (2)	0.0200 (7)	
C9A	0.54827 (18)	0.66957 (18)	0.9473 (2)	0.0207 (7)	
C10A	0.56188 (18)	0.71682 (19)	1.0314 (2)	0.0223 (7)	
H10A	0.6108	0.7051	1.0729	0.033*	
H10B	0.5241	0.7058	1.0614	0.033*	
H10C	0.5596	0.7704	1.0150	0.033*	
N1A	0.58542 (15)	0.67430 (16)	0.8893 (2)	0.0213 (6)	
N2A	0.64370 (16)	0.72396 (16)	0.9091 (2)	0.0211 (6)	
H2NA	0.659 (2)	0.743 (2)	0.9613 (13)	0.025*	
C11A	0.67880 (18)	0.73726 (18)	0.8465 (2)	0.0194 (7)	
S2A	0.75517 (5)	0.79377 (5)	0.87472 (6)	0.02159 (19)	
N3A	0.65198 (17)	0.70631 (19)	0.7655 (2)	0.0261 (6)	
H3N1	0.676 (2)	0.714 (2)	0.729 (2)	0.031*	
H3N2	0.6187 (17)	0.6733 (18)	0.758 (3)	0.031*	
C1B	0.1144 (2)	0.9732 (2)	0.6666 (2)	0.0259 (7)	
C2B	0.1682 (2)	1.0138 (2)	0.7312 (3)	0.0377 (10)	
H2B	0.1676	1.0165	0.7926	0.045*	
C3B	0.2225 (2)	1.0503 (2)	0.7030 (3)	0.0347 (9)	
H3B	0.2594	1.0787	0.7458	0.042*	
C4B	0.2239 (2)	1.0461 (2)	0.6135 (3)	0.0295 (8)	
H4B	0.2614	1.0720	0.5959	0.035*	
C5B	0.1714 (2)	1.0047 (2)	0.5495 (3)	0.0267 (8)	
H5B	0.1730	1.0015	0.4886	0.032*	
C6B	0.11558 (18)	0.96745 (18)	0.5761 (2)	0.0213 (7)	
S1B	0.04048 (5)	0.92419 (5)	0.68465 (6)	0.0294 (2)	
C7B	0.05600 (19)	0.92149 (19)	0.5224 (2)	0.0227 (7)	
H7B	0.0484	0.9107	0.4600	0.027*	
C8B	0.01121 (18)	0.89489 (18)	0.5715 (2)	0.0203 (7)	
C9B	−0.05345 (18)	0.84574 (18)	0.5407 (2)	0.0217 (7)	
C10B	−0.0764 (2)	0.8167 (2)	0.4454 (2)	0.0269 (8)	
H10D	−0.0763	0.7614	0.4461	0.040*	
H10E	−0.0417	0.8349	0.4138	0.040*	
H10F	−0.1262	0.8349	0.4137	0.040*	
N1B	−0.08527 (15)	0.83003 (16)	0.6022 (2)	0.0212 (6)	
N2B	−0.14681 (16)	0.78420 (16)	0.5770 (2)	0.0216 (6)	
H2NB	−0.166 (2)	0.768 (2)	0.5243 (13)	0.026*	
C11B	−0.17783 (18)	0.76294 (19)	0.6424 (2)	0.0202 (7)	
S2B	−0.25346 (5)	0.70679 (5)	0.61499 (6)	0.0227 (2)	
N3B	−0.14553 (16)	0.78669 (17)	0.7263 (2)	0.0233 (6)	
H3N3	−0.1059 (13)	0.811 (2)	0.735 (3)	0.028*	
H3N4	−0.165 (2)	0.770 (2)	0.765 (2)	0.028*	
C1C	0.3583 (2)	0.7371 (2)	0.6860 (2)	0.0258 (7)	
C2C	0.4354 (2)	0.7437 (2)	0.7138 (3)	0.0297 (8)	
H2C	0.4661	0.7002	0.7229	0.036*	
C3C	0.4653 (2)	0.8154 (2)	0.7275 (3)	0.0323 (9)	
H3C	0.5175	0.8214	0.7466	0.039*	
C4C	0.4199 (2)	0.8795 (2)	0.7138 (3)	0.0351 (9)	
H4C	0.4419	0.9281	0.7243	0.042*	
C5C	0.3439 (2)	0.8733 (2)	0.6853 (3)	0.0351 (9)	
H5C	0.3138	0.9172	0.6760	0.042*	
C6C	0.3117 (2)	0.8011 (2)	0.6700 (2)	0.0266 (8)	
S1C	0.30752 (5)	0.65375 (5)	0.66624 (7)	0.0281 (2)	
C7C	0.2349 (2)	0.7803 (2)	0.6387 (3)	0.0287 (8)	
H7C	0.1955	0.8158	0.6234	0.034*	
C8C	0.2248 (2)	0.70426 (19)	0.6336 (2)	0.0244 (7)	
C9C	0.1548 (2)	0.6635 (2)	0.6066 (2)	0.0258 (7)	
C10C	0.0836 (2)	0.7064 (2)	0.5745 (3)	0.0319 (9)	
H10G	0.0759	0.7230	0.5117	0.048*	
H10H	0.0857	0.7506	0.6135	0.048*	
H10I	0.0426	0.6737	0.5773	0.048*	
N1C	0.16139 (17)	0.59049 (17)	0.6156 (2)	0.0297 (7)	
N2C	0.09860 (18)	0.54666 (18)	0.5948 (2)	0.0346 (8)	
H2NC	0.0583 (14)	0.559 (3)	0.556 (3)	0.042*	
C11C	0.1060 (2)	0.4744 (2)	0.6250 (3)	0.0298 (8)	
S2C	0.03402 (6)	0.41383 (6)	0.60223 (8)	0.0448 (3)	
N3C	0.17357 (19)	0.45564 (18)	0.6749 (2)	0.0322 (8)	
H3N5	0.2098 (18)	0.485 (2)	0.688 (3)	0.050 (15)*	
H3N6	0.184 (3)	0.4118 (12)	0.696 (3)	0.051 (15)*	
C1D	0.1294 (2)	0.7575 (2)	0.3394 (3)	0.0292 (8)	
C2D	0.0537 (2)	0.7729 (3)	0.3080 (3)	0.0398 (10)	
H2D	0.0181	0.7340	0.3021	0.048*	
C3D	0.0326 (3)	0.8473 (3)	0.2858 (3)	0.0445 (11)	
H3D	−0.0184	0.8593	0.2636	0.053*	
C4D	0.0844 (3)	0.9040 (3)	0.2953 (3)	0.0412 (10)	
H4D	0.0682	0.9543	0.2798	0.049*	
C5D	0.1592 (2)	0.8893 (2)	0.3268 (3)	0.0347 (9)	
H5D	0.1940	0.9291	0.3334	0.042*	
C6D	0.1832 (2)	0.8146 (2)	0.3492 (2)	0.0277 (8)	
S1D	0.16977 (5)	0.66977 (5)	0.37222 (7)	0.0311 (2)	
C7D	0.2567 (2)	0.7850 (2)	0.3818 (2)	0.0272 (8)	
H7D	0.3000	0.8149	0.3917	0.033*	
C8D	0.2576 (2)	0.7092 (2)	0.3971 (2)	0.0252 (7)	
C9D	0.32188 (19)	0.6605 (2)	0.4331 (2)	0.0245 (7)	
C10D	0.3983 (2)	0.6910 (2)	0.4466 (3)	0.0341 (9)	
H10J	0.4262	0.6885	0.5112	0.051*	
H10K	0.3951	0.7436	0.4261	0.051*	
H10L	0.4234	0.6608	0.4113	0.051*	
N1D	0.30772 (17)	0.59253 (17)	0.4530 (2)	0.0291 (7)	
N2D	0.36755 (19)	0.54664 (19)	0.4909 (3)	0.0376 (8)	
H2ND	0.4088 (15)	0.560 (3)	0.486 (4)	0.060 (17)*	
C11D	0.3565 (2)	0.4790 (2)	0.5254 (3)	0.0313 (8)	
S2D	0.43168 (7)	0.42374 (7)	0.57328 (9)	0.0476 (3)	
N3D	0.28874 (19)	0.45965 (18)	0.5210 (2)	0.0317 (7)	
H3N7	0.2536 (17)	0.489 (2)	0.496 (3)	0.038*	
H3N8	0.284 (3)	0.4170 (13)	0.542 (3)	0.038 (13)*	
O1S	0.1306 (3)	0.4894 (3)	0.4258 (4)	0.0888 (14)	
H1S	0.0944	0.5187	0.4186	0.133*	
C1S	0.1058 (5)	0.4166 (4)	0.4115 (5)	0.087 (2)	
H1S1	0.1150	0.3907	0.4700	0.130*	
H1S2	0.1321	0.3903	0.3743	0.130*	
H1S3	0.0527	0.4166	0.3799	0.130*	
O2S	0.5431 (3)	0.5968 (3)	0.6341 (4)	0.0867 (12)	
H2S	0.5637	0.6279	0.6084	0.130*	
C2S	0.5875 (4)	0.5453 (4)	0.6686 (5)	0.0867 (12)	
H2S1	0.5690	0.4972	0.6390	0.130*	
H2S2	0.5925	0.5417	0.7336	0.130*	
H2S3	0.6357	0.5565	0.6604	0.130*	

### Atomic displacement parameters (Å^2^)

**Table:**

	*U*^11^	*U*^22^	*U*^33^	*U*^12^	*U*^13^	*U*^23^
C1A	0.0172 (16)	0.0190 (15)	0.0232 (16)	0.0036 (12)	0.0044 (13)	0.0020 (13)
C2A	0.0233 (18)	0.0233 (17)	0.0233 (17)	0.0026 (13)	0.0034 (14)	0.0008 (14)
C3A	0.0215 (17)	0.0193 (16)	0.0299 (18)	0.0008 (13)	−0.0009 (14)	0.0008 (14)
C4A	0.0233 (18)	0.0225 (17)	0.0321 (19)	−0.0027 (14)	0.0066 (15)	0.0042 (14)
C5A	0.0221 (17)	0.0248 (17)	0.0305 (18)	−0.0023 (13)	0.0106 (14)	−0.0021 (14)
C6A	0.0184 (16)	0.0195 (15)	0.0250 (17)	0.0019 (13)	0.0051 (13)	0.0008 (13)
S1A	0.0239 (4)	0.0254 (4)	0.0230 (4)	−0.0015 (3)	0.0099 (3)	−0.0025 (3)
C7A	0.0177 (16)	0.0219 (16)	0.0264 (17)	−0.0003 (13)	0.0048 (13)	−0.0024 (14)
C8A	0.0181 (16)	0.0184 (15)	0.0220 (16)	0.0021 (12)	0.0036 (13)	0.0009 (13)
C9A	0.0183 (16)	0.0207 (15)	0.0246 (17)	0.0035 (13)	0.0087 (13)	0.0047 (13)
C10A	0.0161 (16)	0.0240 (16)	0.0264 (17)	−0.0022 (13)	0.0058 (13)	−0.0026 (14)
N1A	0.0154 (13)	0.0234 (14)	0.0245 (14)	−0.0005 (11)	0.0052 (11)	0.0016 (11)
N2A	0.0171 (14)	0.0252 (14)	0.0227 (14)	−0.0016 (11)	0.0084 (11)	0.0004 (12)
C11A	0.0165 (15)	0.0199 (15)	0.0217 (16)	0.0059 (12)	0.0055 (12)	0.0045 (13)
S2A	0.0189 (4)	0.0245 (4)	0.0226 (4)	−0.0021 (3)	0.0080 (3)	0.0015 (3)
N3A	0.0196 (15)	0.0372 (17)	0.0234 (15)	−0.0058 (13)	0.0094 (12)	−0.0016 (13)
C1B	0.0258 (18)	0.0249 (17)	0.0278 (18)	−0.0029 (14)	0.0094 (14)	−0.0011 (14)
C2B	0.041 (2)	0.042 (2)	0.030 (2)	−0.0145 (19)	0.0110 (18)	−0.0093 (18)
C3B	0.030 (2)	0.033 (2)	0.040 (2)	−0.0115 (16)	0.0085 (17)	−0.0105 (17)
C4B	0.0217 (18)	0.0255 (18)	0.044 (2)	−0.0058 (14)	0.0135 (16)	−0.0048 (16)
C5B	0.0233 (18)	0.0245 (17)	0.035 (2)	−0.0019 (14)	0.0126 (15)	−0.0027 (15)
C6B	0.0189 (16)	0.0187 (15)	0.0256 (17)	0.0002 (12)	0.0055 (13)	−0.0002 (13)
S1B	0.0309 (5)	0.0340 (5)	0.0245 (4)	−0.0113 (4)	0.0102 (4)	−0.0034 (4)
C7B	0.0190 (16)	0.0217 (16)	0.0270 (17)	−0.0013 (13)	0.0061 (13)	−0.0008 (14)
C8B	0.0190 (16)	0.0177 (15)	0.0236 (16)	−0.0005 (12)	0.0051 (13)	0.0003 (13)
C9B	0.0186 (16)	0.0203 (16)	0.0256 (17)	0.0032 (13)	0.0056 (13)	0.0015 (13)
C10B	0.0220 (18)	0.0335 (19)	0.0258 (18)	−0.0078 (15)	0.0080 (14)	−0.0050 (15)
N1B	0.0161 (14)	0.0225 (14)	0.0252 (14)	−0.0016 (11)	0.0068 (11)	0.0015 (11)
N2B	0.0172 (14)	0.0250 (14)	0.0227 (14)	−0.0032 (11)	0.0061 (11)	−0.0004 (12)
C11B	0.0160 (15)	0.0221 (16)	0.0228 (16)	0.0048 (12)	0.0062 (12)	0.0022 (13)
S2B	0.0205 (4)	0.0264 (4)	0.0220 (4)	−0.0052 (3)	0.0075 (3)	−0.0001 (3)
N3B	0.0171 (14)	0.0313 (16)	0.0217 (14)	−0.0048 (12)	0.0060 (11)	−0.0023 (12)
C1C	0.0257 (18)	0.0245 (17)	0.0251 (17)	−0.0020 (14)	0.0045 (14)	0.0001 (14)
C2C	0.0250 (19)	0.0319 (19)	0.0292 (19)	−0.0023 (15)	0.0036 (15)	−0.0008 (16)
C3C	0.0253 (19)	0.039 (2)	0.031 (2)	−0.0068 (16)	0.0054 (16)	0.0047 (17)
C4C	0.038 (2)	0.0288 (19)	0.036 (2)	−0.0118 (17)	0.0081 (17)	0.0033 (17)
C5C	0.038 (2)	0.0220 (18)	0.042 (2)	−0.0034 (16)	0.0062 (18)	0.0031 (16)
C6C	0.0262 (19)	0.0250 (17)	0.0275 (18)	−0.0027 (14)	0.0061 (14)	0.0033 (14)
S1C	0.0214 (4)	0.0215 (4)	0.0368 (5)	0.0010 (3)	0.0014 (4)	−0.0024 (4)
C7C	0.0283 (19)	0.0264 (18)	0.0300 (19)	−0.0002 (15)	0.0065 (15)	0.0014 (15)
C8C	0.0240 (18)	0.0226 (17)	0.0244 (17)	0.0028 (14)	0.0035 (14)	0.0041 (14)
C9C	0.0237 (18)	0.0235 (17)	0.0267 (18)	0.0004 (14)	0.0019 (14)	0.0020 (14)
C10C	0.0204 (18)	0.0278 (19)	0.041 (2)	0.0020 (14)	−0.0009 (16)	0.0064 (16)
N1C	0.0202 (15)	0.0247 (15)	0.0375 (18)	−0.0022 (12)	−0.0019 (13)	0.0025 (13)
N2C	0.0241 (17)	0.0233 (15)	0.045 (2)	−0.0010 (13)	−0.0070 (14)	0.0090 (14)
C11C	0.030 (2)	0.0226 (17)	0.0310 (19)	0.0027 (15)	0.0001 (15)	0.0013 (15)
S2C	0.0349 (6)	0.0298 (5)	0.0523 (7)	−0.0090 (4)	−0.0136 (5)	0.0164 (5)
N3C	0.0283 (17)	0.0222 (16)	0.0359 (18)	0.0032 (13)	−0.0060 (14)	0.0041 (14)
C1D	0.028 (2)	0.0323 (19)	0.0278 (18)	0.0034 (15)	0.0088 (15)	0.0008 (15)
C2D	0.027 (2)	0.054 (3)	0.040 (2)	0.0070 (19)	0.0124 (17)	0.008 (2)
C3D	0.036 (2)	0.057 (3)	0.042 (2)	0.021 (2)	0.0135 (19)	0.009 (2)
C4D	0.050 (3)	0.037 (2)	0.043 (2)	0.016 (2)	0.023 (2)	0.0097 (19)
C5D	0.043 (2)	0.0285 (19)	0.036 (2)	0.0057 (17)	0.0164 (18)	0.0034 (17)
C6D	0.030 (2)	0.0279 (18)	0.0256 (18)	0.0026 (15)	0.0094 (15)	0.0015 (15)
S1D	0.0210 (5)	0.0279 (5)	0.0415 (5)	−0.0019 (4)	0.0045 (4)	0.0037 (4)
C7D	0.0275 (19)	0.0286 (18)	0.0246 (18)	0.0000 (14)	0.0064 (14)	0.0024 (14)
C8D	0.0223 (17)	0.0237 (17)	0.0275 (18)	−0.0039 (14)	0.0041 (14)	−0.0005 (14)
C9D	0.0198 (17)	0.0278 (18)	0.0239 (17)	−0.0037 (14)	0.0033 (13)	0.0030 (14)
C10D	0.0218 (19)	0.0304 (19)	0.046 (2)	−0.0019 (15)	0.0044 (17)	0.0099 (18)
N1D	0.0234 (16)	0.0246 (15)	0.0361 (17)	0.0020 (12)	0.0038 (13)	0.0047 (13)
N2D	0.0237 (17)	0.0310 (17)	0.057 (2)	0.0019 (14)	0.0092 (16)	0.0149 (16)
C11D	0.032 (2)	0.0236 (18)	0.036 (2)	0.0032 (15)	0.0071 (16)	0.0038 (16)
S2D	0.0374 (6)	0.0426 (6)	0.0628 (8)	0.0165 (5)	0.0150 (5)	0.0240 (6)
N3D	0.0286 (18)	0.0218 (15)	0.045 (2)	0.0031 (13)	0.0112 (15)	0.0087 (14)
O1S	0.089 (4)	0.094 (4)	0.089 (3)	0.009 (3)	0.035 (3)	−0.003 (3)
C1S	0.111 (6)	0.074 (5)	0.080 (5)	−0.010 (4)	0.034 (4)	−0.018 (4)
O2S	0.099 (3)	0.064 (2)	0.097 (3)	0.013 (2)	0.030 (2)	−0.002 (2)
C2S	0.099 (3)	0.064 (2)	0.097 (3)	0.013 (2)	0.030 (2)	−0.002 (2)

### Geometric parameters (Å, °)

**Table:**

C1A—C2A	1.400 (5)	C3C—H3C	0.9500
C1A—C6A	1.410 (5)	C4C—C5C	1.379 (6)
C1A—S1A	1.737 (3)	C4C—H4C	0.9500
C2A—C3A	1.384 (5)	C5C—C6C	1.406 (5)
C2A—H2A	0.9500	C5C—H5C	0.9500
C3A—C4A	1.400 (5)	C6C—C7C	1.439 (5)
C3A—H3A	0.9500	S1C—C8C	1.744 (4)
C4A—C5A	1.373 (5)	C7C—C8C	1.359 (5)
C4A—H4A	0.9500	C7C—H7C	0.9500
C5A—C6A	1.407 (5)	C8C—C9C	1.458 (5)
C5A—H5A	0.9500	C9C—N1C	1.303 (5)
C6A—C7A	1.434 (5)	C9C—C10C	1.499 (5)
S1A—C8A	1.751 (3)	C10C—H10G	0.9800
C7A—C8A	1.366 (5)	C10C—H10H	0.9800
C7A—H7A	0.9500	C10C—H10I	0.9800
C8A—C9A	1.465 (5)	N1C—N2C	1.376 (4)
C9A—N1A	1.294 (4)	N2C—C11C	1.355 (5)
C9A—C10A	1.501 (5)	N2C—H2NC	0.842 (10)
C10A—H10A	0.9800	C11C—N3C	1.326 (5)
C10A—H10B	0.9800	C11C—S2C	1.687 (4)
C10A—H10C	0.9800	N3C—H3N5	0.842 (10)
N1A—N2A	1.373 (4)	N3C—H3N6	0.840 (10)
N2A—C11A	1.346 (4)	C1D—C2D	1.396 (5)
N2A—H2NA	0.841 (10)	C1D—C6D	1.412 (5)
C11A—N3A	1.319 (4)	C1D—S1D	1.740 (4)
C11A—S2A	1.706 (3)	C2D—C3D	1.390 (6)
N3A—H3N1	0.839 (10)	C2D—H2D	0.9500
N3A—H3N2	0.843 (10)	C3D—C4D	1.382 (7)
C1B—C2B	1.395 (5)	C3D—H3D	0.9500
C1B—C6B	1.405 (5)	C4D—C5D	1.379 (6)
C1B—S1B	1.739 (4)	C4D—H4D	0.9500
C2B—C3B	1.390 (6)	C5D—C6D	1.408 (5)
C2B—H2B	0.9500	C5D—H5D	0.9500
C3B—C4B	1.390 (6)	C6D—C7D	1.432 (5)
C3B—H3B	0.9500	S1D—C8D	1.739 (4)
C4B—C5B	1.386 (5)	S1D—S1B^i^	3.5958 (14)
C4B—H4B	0.9500	C7D—C8D	1.362 (5)
C5B—C6B	1.407 (5)	C7D—H7D	0.9500
C5B—H5B	0.9500	C8D—C9D	1.461 (5)
C6B—C7B	1.438 (5)	C9D—N1D	1.290 (5)
S1B—C8B	1.746 (3)	C9D—C10D	1.500 (5)
C7B—C8B	1.376 (5)	C10D—H10J	0.9800
C7B—H7B	0.9500	C10D—H10K	0.9800
C8B—C9B	1.462 (5)	C10D—H10L	0.9800
C9B—N1B	1.296 (5)	N1D—N2D	1.376 (4)
C9B—C10B	1.495 (5)	N2D—C11D	1.352 (5)
C10B—H10D	0.9800	N2D—H2ND	0.842 (10)
C10B—H10E	0.9800	C11D—N3D	1.311 (5)
C10B—H10F	0.9800	C11D—S2D	1.705 (4)
N1B—N2B	1.379 (4)	N3D—H3N7	0.841 (10)
N2B—C11B	1.362 (4)	N3D—H3N8	0.840 (10)
N2B—H2NB	0.839 (10)	O1S—C1S	1.367 (8)
C11B—N3B	1.325 (4)	O1S—H1S	0.8400
C11B—S2B	1.692 (3)	C1S—H1S1	0.9800
N3B—H3N3	0.838 (10)	C1S—H1S2	0.9800
N3B—H3N4	0.839 (10)	C1S—H1S3	0.9800
C1C—C2C	1.399 (5)	O2S—C2S	1.248 (8)
C1C—C6C	1.413 (5)	O2S—H2S	0.8400
C1C—S1C	1.738 (4)	C2S—H2S1	0.9800
C2C—C3C	1.381 (5)	C2S—H2S2	0.9800
C2C—H2C	0.9500	C2S—H2S3	0.9800
C3C—C4C	1.401 (6)		
			
C2A—C1A—C6A	121.9 (3)	C4C—C3C—H3C	119.5
C2A—C1A—S1A	126.5 (3)	C5C—C4C—C3C	121.3 (4)
C6A—C1A—S1A	111.5 (2)	C5C—C4C—H4C	119.3
C3A—C2A—C1A	117.5 (3)	C3C—C4C—H4C	119.3
C3A—C2A—H2A	121.3	C4C—C5C—C6C	119.0 (4)
C1A—C2A—H2A	121.3	C4C—C5C—H5C	120.5
C2A—C3A—C4A	121.4 (3)	C6C—C5C—H5C	120.5
C2A—C3A—H3A	119.3	C5C—C6C—C1C	118.9 (3)
C4A—C3A—H3A	119.3	C5C—C6C—C7C	129.4 (4)
C5A—C4A—C3A	120.9 (3)	C1C—C6C—C7C	111.7 (3)
C5A—C4A—H4A	119.5	C1C—S1C—C8C	91.05 (17)
C3A—C4A—H4A	119.5	C8C—C7C—C6C	112.6 (3)
C4A—C5A—C6A	119.5 (3)	C8C—C7C—H7C	123.7
C4A—C5A—H5A	120.3	C6C—C7C—H7C	123.7
C6A—C5A—H5A	120.3	C7C—C8C—C9C	127.4 (3)
C5A—C6A—C1A	118.7 (3)	C7C—C8C—S1C	113.1 (3)
C5A—C6A—C7A	129.2 (3)	C9C—C8C—S1C	119.5 (3)
C1A—C6A—C7A	112.1 (3)	N1C—C9C—C8C	114.2 (3)
C1A—S1A—C8A	91.19 (16)	N1C—C9C—C10C	125.9 (3)
C8A—C7A—C6A	112.7 (3)	C8C—C9C—C10C	119.8 (3)
C8A—C7A—H7A	123.6	C9C—C10C—H10G	109.5
C6A—C7A—H7A	123.6	C9C—C10C—H10H	109.5
C7A—C8A—C9A	128.3 (3)	H10G—C10C—H10H	109.5
C7A—C8A—S1A	112.5 (3)	C9C—C10C—H10I	109.5
C9A—C8A—S1A	119.1 (3)	H10G—C10C—H10I	109.5
N1A—C9A—C8A	114.7 (3)	H10H—C10C—H10I	109.5
N1A—C9A—C10A	124.9 (3)	C9C—N1C—N2C	119.1 (3)
C8A—C9A—C10A	120.4 (3)	C11C—N2C—N1C	117.3 (3)
C9A—C10A—H10A	109.5	C11C—N2C—H2NC	117 (3)
C9A—C10A—H10B	109.5	N1C—N2C—H2NC	124 (3)
H10A—C10A—H10B	109.5	N3C—C11C—N2C	114.8 (3)
C9A—C10A—H10C	109.5	N3C—C11C—S2C	122.9 (3)
H10A—C10A—H10C	109.5	N2C—C11C—S2C	122.3 (3)
H10B—C10A—H10C	109.5	C11C—N3C—H3N5	124 (4)
C9A—N1A—N2A	117.0 (3)	C11C—N3C—H3N6	122 (4)
C11A—N2A—N1A	119.2 (3)	H3N5—N3C—H3N6	114 (5)
C11A—N2A—H2NA	120 (3)	C2D—C1D—C6D	122.1 (4)
N1A—N2A—H2NA	120 (3)	C2D—C1D—S1D	126.4 (3)
N3A—C11A—N2A	118.2 (3)	C6D—C1D—S1D	111.5 (3)
N3A—C11A—S2A	123.0 (3)	C3D—C2D—C1D	117.4 (4)
N2A—C11A—S2A	118.8 (3)	C3D—C2D—H2D	121.3
C11A—N3A—H3N1	116 (3)	C1D—C2D—H2D	121.3
C11A—N3A—H3N2	119 (3)	C4D—C3D—C2D	121.3 (4)
H3N1—N3A—H3N2	124 (4)	C4D—C3D—H3D	119.3
C2B—C1B—C6B	121.5 (3)	C2D—C3D—H3D	119.3
C2B—C1B—S1B	126.5 (3)	C5D—C4D—C3D	121.5 (4)
C6B—C1B—S1B	111.9 (3)	C5D—C4D—H4D	119.2
C3B—C2B—C1B	117.9 (4)	C3D—C4D—H4D	119.2
C3B—C2B—H2B	121.0	C4D—C5D—C6D	119.2 (4)
C1B—C2B—H2B	121.0	C4D—C5D—H5D	120.4
C2B—C3B—C4B	121.3 (4)	C6D—C5D—H5D	120.4
C2B—C3B—H3B	119.4	C5D—C6D—C1D	118.4 (4)
C4B—C3B—H3B	119.4	C5D—C6D—C7D	129.8 (4)
C5B—C4B—C3B	120.9 (4)	C1D—C6D—C7D	111.8 (3)
C5B—C4B—H4B	119.5	C8D—S1D—C1D	90.87 (18)
C3B—C4B—H4B	119.5	C8D—S1D—S1B^i^	137.93 (13)
C4B—C5B—C6B	118.9 (4)	C1D—S1D—S1B^i^	92.50 (13)
C4B—C5B—H5B	120.5	C8D—C7D—C6D	112.5 (3)
C6B—C5B—H5B	120.5	C8D—C7D—H7D	123.8
C1B—C6B—C5B	119.3 (3)	C6D—C7D—H7D	123.8
C1B—C6B—C7B	111.8 (3)	C7D—C8D—C9D	127.8 (3)
C5B—C6B—C7B	128.9 (3)	C7D—C8D—S1D	113.3 (3)
C1B—S1B—C8B	91.17 (17)	C9D—C8D—S1D	118.8 (3)
C8B—C7B—C6B	112.6 (3)	N1D—C9D—C8D	115.6 (3)
C8B—C7B—H7B	123.7	N1D—C9D—C10D	124.3 (3)
C6B—C7B—H7B	123.7	C8D—C9D—C10D	120.1 (3)
C7B—C8B—C9B	128.3 (3)	C9D—C10D—H10J	109.5
C7B—C8B—S1B	112.5 (3)	C9D—C10D—H10K	109.5
C9B—C8B—S1B	119.2 (3)	H10J—C10D—H10K	109.5
N1B—C9B—C8B	114.7 (3)	C9D—C10D—H10L	109.5
N1B—C9B—C10B	125.7 (3)	H10J—C10D—H10L	109.5
C8B—C9B—C10B	119.7 (3)	H10K—C10D—H10L	109.5
C9B—C10B—H10D	109.5	C9D—N1D—N2D	116.7 (3)
C9B—C10B—H10E	109.5	C11D—N2D—N1D	119.1 (3)
H10D—C10B—H10E	109.5	C11D—N2D—H2ND	123 (4)
C9B—C10B—H10F	109.5	N1D—N2D—H2ND	117 (4)
H10D—C10B—H10F	109.5	N3D—C11D—N2D	118.3 (3)
H10E—C10B—H10F	109.5	N3D—C11D—S2D	123.5 (3)
C9B—N1B—N2B	117.2 (3)	N2D—C11D—S2D	118.2 (3)
C11B—N2B—N1B	117.9 (3)	C11D—N3D—H3N7	120 (3)
C11B—N2B—H2NB	118 (3)	C11D—N3D—H3N8	116 (3)
N1B—N2B—H2NB	125 (3)	H3N7—N3D—H3N8	124 (5)
N3B—C11B—N2B	117.4 (3)	C1S—O1S—H1S	109.5
N3B—C11B—S2B	122.8 (3)	O1S—C1S—H1S1	109.5
N2B—C11B—S2B	119.8 (3)	O1S—C1S—H1S2	109.5
C11B—N3B—H3N3	116 (3)	H1S1—C1S—H1S2	109.5
C11B—N3B—H3N4	115 (3)	O1S—C1S—H1S3	109.5
H3N3—N3B—H3N4	129 (4)	H1S1—C1S—H1S3	109.5
C2C—C1C—C6C	121.8 (3)	H1S2—C1S—H1S3	109.5
C2C—C1C—S1C	126.7 (3)	C2S—O2S—H2S	109.5
C6C—C1C—S1C	111.5 (3)	O2S—C2S—H2S1	109.5
C3C—C2C—C1C	117.9 (4)	O2S—C2S—H2S2	109.5
C3C—C2C—H2C	121.1	H2S1—C2S—H2S2	109.5
C1C—C2C—H2C	121.1	O2S—C2S—H2S3	109.5
C2C—C3C—C4C	121.0 (4)	H2S1—C2S—H2S3	109.5
C2C—C3C—H3C	119.5	H2S2—C2S—H2S3	109.5

Symmetry codes: (i) *x*, −*y*+3/2, *z*−1/2.

### Hydrogen-bond geometry (Å, °)

**Table:**

*D*—H···*A*	*D*—H	H···*A*	*D*···*A*	*D*—H···*A*
N3A—H3N2···N1A	0.843 (10)	2.29 (4)	2.641 (4)	105 (3)
N3B—H3N3···N1B	0.838 (10)	2.21 (4)	2.617 (4)	110 (3)
N3C—H3N5···N1C	0.842 (10)	2.22 (5)	2.543 (4)	102 (4)
N3D—H3N7···N1D	0.841 (10)	2.29 (5)	2.643 (4)	105 (4)
N3A—H3N1···S2B^ii^	0.839 (10)	2.500 (14)	3.325 (3)	168 (4)
N3B—H3N4···S2A^iii^	0.839 (10)	2.62 (2)	3.367 (3)	149 (4)
N2B—H2NB···S2A^iv^	0.839 (10)	2.585 (13)	3.412 (3)	169 (4)
N3C—H3N6···S2A^v^	0.840 (10)	2.76 (4)	3.352 (3)	129 (4)
N3C—H3N6···N3B^vi^	0.840 (10)	2.72 (3)	3.468 (5)	149 (5)
N2C—H2NC···S2C^vii^	0.842 (10)	2.60 (2)	3.392 (3)	158 (4)
N3D—H3N8···S2A^v^	0.840 (10)	2.738 (15)	3.563 (3)	168 (4)
N3D—H3N7···O1S	0.841 (10)	2.26 (3)	2.967 (6)	142 (4)
O1S—H1S···S2C^vii^	0.84	2.64	3.471 (5)	169
O2S—H2S···S2D^viii^	0.84	2.97	3.389 (6)	113
C3A—H3A···S2A^v^	0.95	2.97	3.762 (4)	142
C10A—H10A···S2B^ix^	0.98	2.91	3.613 (3)	129
C2B—H2B···O1S^x^	0.95	2.36	3.282 (7)	163
C2S—H2S1···N2D^viii^	0.98	2.74	3.266 (9)	115
C10B—H10F···C_g_^xi^	0.98	2.91	3.594 (4)	129

Symmetry codes: (ii) *x*+1, *y*, *z*; (iii) *x*−1, *y*, *z*; (iv) *x*−1, −*y*+3/2, *z*−1/2; (v) −*x*+1, *y*−1/2, −*z*+3/2; (vi) −*x*, *y*−1/2, −*z*+3/2; (vii) −*x*, −*y*+1, −*z*+1; (viii) −*x*+1, −*y*+1, −*z*+1; (ix) *x*+1, −*y*+3/2, *z*+1/2; (x) *x*, −*y*+3/2, *z*+1/2; (xi) −*x*, −*y*+1, −*z*.
